# Oral health and the impact of socio-behavioural factors in a cross sectional survey of 12-year old school children in Laos

**DOI:** 10.1186/1472-6831-9-29

**Published:** 2009-11-16

**Authors:** Nanna Jürgensen, Poul Erik Petersen

**Affiliations:** 1 Department for Community Dentistry and Graduate Studies, Faculty of Health Sciences, University of Copenhagen, Oester Farimagsgade 5, DK - 1014 Copenhagen K, Denmark; 2 World Health Organization, Chronic Disease and Health Promotion, 20 Ave Appia, CH1211 Geneva Switzerland

## Abstract

**Background:**

In recent decades low-income countries experienced an increasing trend in dental caries among children, particularly recorded in 12-year olds, which is the principal WHO indicator age group for children. This increases the risks of negative affects on children's life. Some data exist on the oral health status of children in low-income countries of Southeast Asia. However, information on how oral health is associated with socio-behavioural factors is almost not available. The aims of this study were to: assess the level of oral health of Lao 12-year-olds in urban and semi-urban settings; study the impact of poor oral health on quality of life; analyse the association between oral health and socio-behavioural factors; investigate the relation between obesity and oral health.

**Methods:**

A cross sectional study of 12-year old schoolchildren chosen by multistage random sampling in Vientiane, Lao P.D.R (hereafter Laos). The final study population comprised 621 children. The study consisted of: clinical registration of caries and periodontal status, and scores for dental trauma according to WHO; structured questionnaire; measurement of anthropometric data. Frequency distributions for bi-variate analysis and logistic regression for multivariate analysis were used for assessment of statistical association between variables.

**Results:**

Mean DMFT was 1.8 (SEM = 0.09) while caries prevalence was 56% (CI95 = 52-60). Prevalence of gingival bleeding was 99% (CI95 = 98-100) with 47% (CI95 = 45-49) of present teeth affected. Trauma was observed in 7% (CI95 = 5-9) of the children. High decay was seen in children with dental visits and frequent consumption of sweet drinks. Missed school classes, tooth ache and several impairments of daily life activities were associated with a high dD-component. No associations were found between Body Mass Index (BMI) and oral health or common risk factors. The multivariate analyses revealed high risk for caries for children with low or moderate attitude towards health, a history of dental visits and a preference for drinking sugary drinks during school hours. Low risk was found for children with good or average perception of own oral health. High risk for gingival bleeding was seen in semi-urban children and boys.

**Conclusion:**

Although the caries level is low it causes considerable negative impact on daily life. School based health promotion should be implemented focussing on skills based learning and attitudes towards health.

## Background

Historically diseases of the oral cavity have been viewed separately from those of the rest of the body. In recent years however efforts have been made to recognize oral health as an integral part of overall health [1]. Moreover, the oral cavity has a multitude of functions in relation to daily life such as food intake, speech, social contact and appearance. Poor oral health has thus the potential of hampering the quality of life. Decreased food intake because of oral pain or poor dental status can cause low growth in children [2] and may worsen the nutritional status. Pain might also have a negative impact on the ability to engage in social relations and children might not get the full benefit of their education if suffering from pain and discomfort. While poor dental status among children has a negative effect on speech development, it may also have a socially stigmatising effect in adolescents affecting social acceptance [3].

Dental caries is the most prevalent chronic disease among children worldwide. While dental caries has decreased in many industrialised countries the contrary is the case in many low-income countries [4]. The increase of caries is caused by a variety of factors one being the adoption of food habits high in refined carbohydrates. Health behaviour such as the use of fluoridated tooth paste and regular tooth brushing is rare among children in low-income countries [3,5,6]. In addition, national public health programmes often do not consider oral health.

Oral epidemiological information is available from studies carried out in South East Asian countries, e.g. China and Thailand, and low to moderate caries levels among children are revealed. Petersen et al [7] found a mean DMFT of 2.4 in a Thai study of 12-year-olds while surveys of the same age group of children in China observed a rather low mean DMFT of 0.8-1.0 [8-10]. A national oral health survey among 12-year-olds was undertaken in Laos in 1991 [11]. The mean DMFT was 2.0 ranging from 1.1 to 3.9 while the mean percentage of caries free children of this age was 40%. Since 1991, only minor studies have been implemented locally. However, an increase in the availability of refined foods high in sugar has been noticed for Laos over the past years. This is evidenced by data from the Sugar Year Book, which shows an increase in sugars consumption for the country from 1.7 kg/year/person in 1991 to 7.6 kg/person/year in 2005 [12].

The aim of this study is to describe the oral health status of 12-year old urban and semi-urban Lao schoolchildren and how oral health is linked to socio-behavioural risk factors. Furthermore, the aim is to ascertain the impact of poor oral health on the quality of life of children in a low-income country, to analyse the association of socio-demographic and behavioural factors with oral health and to investigate possible relationships between obesity and oral health and risk factors for chronic disease. These aspects are crucial to oral health promotion and integration of oral disease prevention into the Lao school health initiative.

## Methods

According to the 2007/08 Human Development Index Laos is ranked number 130 out of 177 countries. For the population of 5.6 million oral health care is provided by a limited number of dentists [11] mainly operating from dental clinics at central or provincial levels and at certain district hospitals. In addition, a number of private dental clinics operate after official working hours primarily in the capital of Vientiane and in the larger urban settlements. In rural areas access to dental care is limited if present at all. Oral health is so far not officially represented within the Lao Ministry of Health. It is however included in the national school curriculum although with a rather biomedical approach. A national school health partnership has been established jointly by the Ministries of Health and Education initially to fight soil transmitted helminths with health promotion and treatment in primary schools. Automatic fluoridation is not introduced in Laos.

This study was carried out in September and October 2006 in Vientiane as a cross sectional survey of 12-year old schoolchildren. The sample size was estimated from information on dental caries prevalence parameters observed in previous epidemiological studies carried out in Laos. Multistage sampling was applied to ensure that the final study population would match the categories mentioned in the objective of the survey. Ten secondary schools were selected to represent schools of urban and semi-urban settings. Of each school, 2^nd ^grade classes were randomly chosen and all 11-13 year old children in the selected classes were asked to participate. According to information from the educational authorities, enrolment rates for schools eligible to participate in the study were above 90% for both sexes. Parents and children were informed about the study and aware of the fact that the participants could withdraw from the study at any time. The response rate was 94% and the final sample comprised of 621 children. The schools were visited at least twice and no further attempt was made to include non-attendants.

The survey involved a structured self-administered questionnaire followed by an oral examination, and measurements of height and weight. The completion of the questionnaire and the clinical examination were conducted at the same day. The clinical examination assessed the dentition status, gingival health and dental trauma and was based on the methods and criteria described by the World Health Organization (WHO) [13]. Dental caries was recorded at the cavity level in order to measure the caries experience indices (dmft/DMFT). Dental trauma was recorded if signs of treated or untreated fracture, discoloration, fistula or a missing tooth were present. Finally the Community Periodontal Index (CPI) was applied to describe the gingival health conditions. The conditions recorded included CPI-scores 0 (healthy) and 1 (bleeding). As recommended by the WHO, periodontal pockets were not recorded as the survey population was under the age of 15 years. Visual inspection was used for registration of dental caries and the clinical examinations were carried out in daylight using a plane mouth mirror. The CPI probe was used for assessment of gingival conditions [13].

An international oral epidemiologist (PEP) ensured calibration of the clinical examiners in Laos (NJ and SAY) through duplicate caries examinations of children aged 7-13 not included in the final survey (n = 14). The inter-examiner agreement with regards to caries diagnosis was expressed by the percentage agreement and by the Kappa statistics as recommended by the WHO [14]. Kappa statistics at the level 0.88 - 0.91 were achieved. No attempt was made to assess the inter-examiner agreement as regards gingival conditions due to the nature of gingival disease. However, criteria and examinations were discussed carefully. An additional calibration trial involving the principal investigator (NJ) and a local dentist (SAC) assigned to the survey took place. Kappa statistics at the level 0.87 was achieved. Halfway through the survey the dental examiners undertook a second calibration trial and Kappa statistics was 0.88.

All children taking part in the study were invited to complete a structured questionnaire on socio-behavioural risk factors. The following principal variables were covered: knowledge, attitudes and practices related to oral health; dietary habits; self-assessment of oral and general health status; experience of pain and discomfort; patterns of physical exercise; residential area; and socio-economic situation. Oral health knowledge and attitudes were measured by use of standardised questions and by positive or negative responses to loaded statements [15]. The children were asked for the brand name of the toothpaste they currently used. Information on fluoride content was afterwards collected form the content declarations. The construction of questionnaires was based on experiences gained from surveys carried out by the WHO Collaborating Centre for Community Oral Health Programmes and Research, University of Copenhagen http://www.who.int/oral_health. The questionnaires were formulated in English, translated into Lao and pre-tested among children not included in the study in order to control the reliability and validity [16]. The research study was approved by the Ethical Committee of the Faculty of Medical Sciences, National University of Laos and informed consent was obtained from the participants.

As part of the clinical examination, all children had their weight and height measured to be able to calculate their body mass index (BMI). Measurements were taken with the children wearing school uniform and no shoes. The height was measured with the child standing adjacent to a wooden pole with an attached centimetre band. Height was measured in full centimetres. The weight was measured on a mechanical weight scale in half or full kilograms. To group the children according to BMI two methods were applied; one using cut points for normal weight and overweight elaborated by TJ Cole on the basis of international data including Asians [17]; and a second empirical method by dividing the children into tertiles. As part of the sociological component of the study [16] several additive indices were constructed to measure socio-economic background, dietary habits, knowledge about items harmful to oral health, and attitude to health. The indices were categorised into three or five levels on the basis of the empirical distributions of the sum scores [16].

Data processing, data analyses and statistical evaluation were performed by means of the Statistical Package for the Social Sciences, SPSS 14.0 and 16.0. The data were described by uni- and bivariate frequency distributions and the prevalence proportion rates of dental caries and gingival bleeding were calculated. Moreover, the mean dmft-DMFT indices and the mean proportion of teeth with gingival bleeding were computed. The statistical evaluation of means was performed by Students t-test where the means of two groups were compared and by ANOVA where the means of more than two groups were compared. The Chi square test was used to compare proportions. In order to assess the relative effect of socio-demographic and behavioural factors on dental caries multiple linear regression and logistic regression analysis were carried out. In the logistic regression analyses presence or absence of caries was used as the dependent variable and the regression coefficient was the odds ratio for caries (OR = P/1-P). The coefficient was tested statistically by the Wald Chi Square test. In the linear regression analyses the caries indices were used as dependent variables and the t-test was used for statistical evaluation of regression coefficients.

## Results

### Oral health status

The total dental caries prevalence (dmft-DMFT) among the children is shown in Table 1. The mean DMFT was 1.8 (SEM = 0.09) (Table 2) while mean dmft was 0.4 (SEM = 0.04). For both dentitions untreated caries was dominant. Somewhat higher caries experience was seen in children of privileged economic background than poor (Table 2). Children from semi-urban areas had significantly higher dD-component compared to urban children while fillings were more frequently seen among children living in urban areas, those coming from privileged socio-economic background or having a literate mother. Fissure sealant was only registered in one child. The overall prevalence of gingival bleeding was 99% (CI95 = 98-100) and the proportion of teeth affected with gingival bleeding was 47% (CI95 = 45-49). Boys, children living in semi-urban areas and children with disadvantaged socio-economic background had significantly higher proportion of teeth with gingival bleeding.

**Table 1:** Prevalence proportion (%) of caries indices, gingival bleeding and dental trauma by gender and socio-demographic factors (CI95 in brackets)

	n	PP of dD	PP of mM	PP of fF	PP of DMFT	PP of dmft-DMFT	PP of gingival bleeding	PP of trauma
**Total** **(CI95)**	621	61.4 (58-65)	14.2 (11-17)	7.4 (5-7)	56.2 (52-60)	65.4 (62-69)	98.7 (98-100)	7.2 (5-9)

**Gender**								
Boys	293	60.4	14.0	5.5	52.6	62.8	99.0	11.6***
Girls	328	62.2	14.3	9.1	59.5	67.7	98.5	3.4

**Mothers literacy**								
Illiterate	143	60.8	9.8	1.4	51.0	62.2	99.3	8.4
Literate	469	61.2	15.6	9.0**	57.4	65.9	98.5	6.8

**Urban location**								
Urban	297	56.9	16.2	10.8**	52.2	63.3	98.0	7.1
Semi urban	324	65.4*	12.3	4.3	59.9*	67.3	99.4	7.4

**Economic background**								
Very low	115	53.9	7.8	2.6	45.2	56.5	100.0	6.1
Low	117	59.8	18.8	3.4	52.1	63.2	98.3	7.7
Medium	130	66.2	9.2	3.1	61.5	68.5	99.2	7.7
High	136	66.2	20.6**	11.0	61.8*	69.9	99.3	5.9
Very high	123	59.3	13.8	16.3 ***	58.5	67.5	96.7	8.9

* p < 0.05 **p < 0.01***p < 0.001

**Table 2:** Mean caries indices and proportion of teeth with gingival bleeding among 12 year olds in relation to socio-demographic factors (SEM or CI95 in brackets)

	n	dDt	mMt	fFt	DMFT	dmft-DMFT	Proportion of teeth with gingival bleeding
**Total** **(SEM)** **(CI95)**	621	1.8 (0.08)	0.2 (0.03)	0.2 (0.03)	1.8 (0.09)	2.2 (0.10)	46.9 (44.8-48.9)

**Gender**							
Boys	293	1.6	0.3	0.1	1.5	2.0	50.1**
Girls	328	1.9	0.2	0.2	2.0**	2.3	44.0

**Urban location**							
Urban	297	1.6	0.3	0.2**	1.6	2.1	41.7
Semi-urban	324	2.0**	0.2	0.1	1.9	2.3	51.6***

**Mothers literacy**							
Illiterate	143	1.8	0.2	0.0	1.6	2.0	48.5
Literate	469	1.8	0.3	0.2**	1.8	2.2	46.0

**Economic background**							
Very low	115	1.5	0.2	0.0	1.2	1.7	52.1***
Low	117	1.8	0.3	0.0	1.6	2.2	49.2
Medium	130	2.0	0.2	0.1	1.9	2.2	48.0
High	136	2.1	0.3	0.2	2.2*	2.6*	47.0
Very high	123	1.5	0.3	0.4***	1.8	2.2	38.3

* p < 0.05 **p < 0.01 ***p < 0.001

Seven percent of the children had signs of dental trauma (CI95 = 5-9) (Table 1), boys showing higher prevalence of trauma than girls (p < 0.001). Restoration of traumatised teeth was only observed in 7% of the children with dental trauma. While 43% of all traumas had occurred during playtime, 21% of the incidents occurred during sports activities. Traffic accidents were mentioned as the reason for 9% of dental trauma cases while 27% of the children either mentioned "other reasons" or could not remember the cause of the trauma.

### Dental caries and own perception of oral and general health

Caries experience was highly associated with perceived oral health status, especially in regard to the dD-component (Table 3). No significant associations were found between gingival status and perception of oral health. Total caries experience decreased with increased perception of good general health; meanwhile, this finding was not statistically significant. Significantly higher mean number of missing teeth was however seen among children who rated their general health to be poor.

**Table 3:** Mean caries indices in relation to self-assessment of oral and general health status, experience of toothache, and absenteeism from school

	n	dDT	mMT	fFT	dmft-DMFT
**Self-assessment of oral health**					

Good	83	1.2	0.2	0.2	1.6

Average	343	1.6	0.2	0.1	1.9

Poor	112	2.7***	0.3	0.1	3.2***

Don't know	82	1.9	0.3	0.3	2.5

Total	620	1.8	0.2	0.2	2.2

**Self-assessment of general health**					

Very healthy	45	1.4	0.1	0.1	1.5

Quite healthy	365	1.8	0.3	0.2	2.2

Not very healthy	56	2.1	0.2	0.1	2.4

Poor health	11	2.4	0.7*	0.0	3.1

Don't know	143	1.7	0.2	0.2	2.0

Total	620	1.8	0.2	0.2	2.2

**Experience of tooth ache in previous 12 months**					

Often	132	2.4***	0.3	0.2	2.9***

Rarely	280	1.9	0.3	0.1	2.3

Never	182	1.2	0.2	0.2	1.5

Total	594	1.8	0.2	0.1	2.2

**Absenteeism from school in previous 12 months**					

Several times	57	2.6***	0.4	0.1	3.0**

Once	176	2.0	0.3	0.1	2.4

Never	388	1.6	0.2	0.2	2.0

Total	621	1.8	0.2	0.2	2.2

*p < 0.05 **p < 0.01 ***p < 0.001

### Oral health related problems and daily life activities

Table 3 shows caries indices for certain frequencies of tooth ache and absenteeism form school due to tooth ache. The children experiencing frequent tooth ache or stating several episodes of absenteeism had a significantly higher dD-component and total caries experience compared to their peers. High caries indices were also found for children with impairment of quality of life such as problems with eating, smiling and sleeping (Table 4).

**Table 4:** Daily activities affected by tooth problems and significant findings in associated caries indices

Quality of life dimension	n	Problems encountered with activity or not	dDT	mMT	fFT	dmft-DMFT
**Eating**	289	Problem	2.2***	0.3**	0.1	2.7***
	332	No problem	1.4	0.2	0.2	1.7

**Smiling**	90	Problem	2.3*	0.3	0.2	2.8**
	531	No problem	1.7	0.2	0.1	2.1

**Sleeping**	67	Problem	2.3*	0.4	0.3	2.9**
	554	No problem	1.7	0.2	0.1	2.1

*p < 0.05 **p < 0.01 ***p < 0.001

### Dental visits

As shown in Table 5, the mean caries experience of the children with a history of recent dental visit was significantly higher compared to children, who had never been to the dentist. All caries components were higher among the children who had seen a dentist compared to those who had never seen a dentist. These differences were highly significant for missing and filled teeth. Although untreated caries was higher in children having seen a dentist, the difference compared to the children with rare or no dental visits was not statistically significant. The total caries experience for children with planned versus acute dental visits were 2.1 versus 2.8 (p < 0.05). No difference in gingival health was observed across dental visit habits.

**Table 5:** Mean caries indices and caries prevalence proportion (%) according to dental visits, tooth brushing and drinks consumed at school

Dental visits	n	dDt	mMt	fFt	dmft-DMFT	PP of dmft-DMFT
**Time since last visit**						

Within the last 12 months	180	1.9	0.4**	0.3***	2.6**	71.7*
More than 12 months ago	177	2.0	0.3	0.1	2.4	72.9
Never been to a dentist	262	1.6	0.1	0.0	1.8	55.7
Total	619	1.8	0.2	0.2	2.2	65.3

**Type of visits**						

Planned visits	90	1.6	0.3	0.24	2.1	68.9
Acute visits	154	2.2*	0.4	0.25	2.8*	76.0
Total	244	2.0	0.4	0.25	2.6	73.4

**Tooth brushing habits**						

Once daily or less	142	2.1*	0.1	0.0	2.3	68.3
Twice daily or more	474	1.7	0.3*	0.2*	2.2	64.8
Total	616	1.8	0.2	0.2	2.2	65.6

**Most consumed drink during school hours**						

Non sugary drinks	319	1.6	0.2	0.2	1.9	58.9
Sugary drinks	298	2.0*	0.3	0.2	2.4*	72.1**
Total	617	1.8	0.2	0.2	2.2	65.3

*p < 0.05 **p < 0.01 ***p < 0.001

### Oral hygiene behaviours and sugar consumption

Of all children 91% reported the use of fluoridated toothpaste but differences in caries level could not be observed between users of fluoridated and non-fluoridated toothpaste. In children who stated tooth brushing twice daily or more a low dD-component was seen (Table 5). The mM- and fF-components were however higher compared to the children brushing their teeth less frequent. No significant difference by oral hygiene habits was seen for the gingival status. Children with a preference for intake of sweet drinks at school had a high mean dD-component and a high dmft-DMFT compared to children drinking mostly water. No differences in caries levels were observed in relation to consumption of sugary or healthy foods or the frequency of which sugary items were eaten.

### Health related attitude and oral health related knowledge

With respect to the oral health knowledge indices no significant differences in caries level were found. Meanwhile, the pattern for children's attitude towards health was different (Table 6). While the total caries experience among children with moderate attitude was 2.5 it was only 1.7 among children with high attitude towards health. Total caries prevalence was 70% versus 56%, respectively. The same trend was seen for gingival bleeding although differences here were not statistically significant.

**Table 6:** Mean caries indices, caries prevalence (%) and proportion of teeth with gingival bleeding by attitude towards health (n = 621)

	dD-T	mM-T	fF-T	dmft/DMFT	PP dmft/DMFT	Proportion of teeth with gingival bleeding
**Attitude level towards health**						

Low	1.9	0.3	0.1	2.2	70.1**	49.6
Moderate	2.0*	0.3*	0.2	2.5**	69.8	46.7
High	1.4	0.1	0.2	1.7	55.9	43.6
Total	1.8	0.2	0.2	2.1	65.8	46.8

*p < 0.05 **p < 0.01

### BMI and oral health

Applying the cut-points suggested by TJ Cole 60% of the children had a normal BMI while 32% were under- and 8% overweight. Significantly more overweight children were observed among urban children compared to semi-urban (12% versus 5%, p < 0.01). Overweight was found especially in the socio-economic advantaged group of children (14%) compared to the disadvantaged group (4%) although findings were not statistically significant. Similar results were found after dividing BMI scores into tertiles. The highest caries level (dmft-DMFT = 2.3) was found among children with normal weight while overweight children had the lowest caries level (1.6) in both methods used.

### Multivariate analyses

Other factors being equal, the analyses revealed a relatively high dental caries score in children who reported dental visits in recent 12 months and in children with a moderate attitude towards health while a lower caries level was seen among children who perceived their own oral health status as good or average (Table 7). High odds (OR) for dental caries was seen among children with low or moderate attitudes towards health, for children with a history of dental visits and children favouring sugary drinks while children who perceived their oral health being good or average had a lower caries risk. Other factors being equal, a relatively high proportion of teeth with gingival bleeding was observed among boys and children from semi-urban settings.

**Table 7:** Multivariate regression analyses of dependant variables mean number of teeth with bleeding gums, mean caries components (b) and total caries experience (OR) by socio-demographic and behavioural factors (n = 621)

Independent variable	Category	Number of teeth with gingival bleeding (b)	dDT (b)	mMT (b)	fFT (b)	dmft-DMFT (b)	Caries (OR)
**Gender**	Boys	1.48*	-0.25	0.07	-0.09	-0.28	0.82
	Girls	-	-	-	-	-	-

**Location**	Semi-urban	2.25**	0.30	-0.16*	-0.06	0.08	1.16
	Urban	-	-	-	-	-	-

**Socio-economic position**	Very low	1.97	-0.14	0.04	-0.21*	-0.32	0.78
	Low	1.83	-0.03	0.09	-0.20*	-0.14	0.71
	Moderate	0.95	0.09	-0.08	-0.21*	-0.20	0.84
	High	1.37	0.50	0.11	-0.10	0.51	1.41
	Very high	-	-	-	-	-	-

**Literacy of mother**	Literate	0.02	0.14	0.08	0.05	0.27	1.20
	Illiterate	-	-	-	-	-	-

**Attitude towards health**	Low	0.21	0.19	0.23**	0.01	0.43	1.79*
	Moderate	0.06	0.50*	0.22**	0.04	0.76**	1.99**
	High	-	-	-	-	-	-

**Dental visits**	Within last 12 months	-0.08	0.29	0.17*	0.20**	0.65**	2.11**
	More than 12 months ago	-1.15	0.09	0.12	0.03	0.24	1.82*
	Never	-	-	-	-	-	-

**Favourite drink**	Sugary	0.18	0.22	0.09	-0.03	0.27	1.53*
	Non-sugary	-	-	-	-	-	-

**Self-assessment of oral health**	Good	-0.78	-1.52***	-0.19	0.02	-1.69***	0.24***
	Average	-0.84	-1.19***	-0.15	0.00	-1.35***	0.39**
	Poor	-	-	-	-	-	-

**Tooth brushing**	At least twice daily	0.44	-0.21	0.18*	0.04	0.02	0.94
	Once or less often	-	-	-	-	-	-

**R^2^**		0.07	0.12	0.07	0.06	0.12	0.14

*p < 0.05 **p < 0.01 ***p < 0.001

## Discussion

In Laos systematic information about the oral health situation is scarce. Regular data collection does not take place and the only national oral health survey was implemented back in 1991. The present study provides information on the oral health status for urban and semi-urban 12-year old schoolchildren in Vientiane. The survey includes important target groups where it is expected that the oral disease pattern would reflect the changing living conditions and adoption of modern lifestyles. The study focuses on two of the most common chronic oral diseases in this age group, i.e. dental caries and gingival problems. As living conditions, lifestyles, and morbidity profiles are markedly different in urban and rural areas of Laos, this survey cannot be seen as representative for the entire country. It has however most likely relevance to children in and around the larger urban settlements of Laos.

In many high-income countries, research can benefit from a number of conditions of importance to sampling of a study population. For example, established population registries provide an opportunity of probability sampling. Furthermore, longitudinal data bases make it possible in studies of health and illness directly to assess the disease risks (RR), while in cross-sectional surveys it is needed to estimate the risk in terms of the Odds Ratio. For example, in Denmark, 98% of the child population and young people up to the age of 18 are covered by the public dental service and a unique population database exists as the children are examined on a regular basis. In low-income countries, such as Laos, however, alternative ways are to be identified in order to select a relevant sample for a survey. The sample for the present study was drawn from a list of well-defined secondary schools and these settings provide for a public health relevant sample and a high number of study units of the age group of interest. Moreover, this selection method allows for the data to be acquired in a timely manner assuring a high response rate at the same time. Nevertheless several aspects should be born in mind when using this approach. Firstly, risk of selection bias is present if children in high numbers are not attending school. The non-attendees are likely to represent a deprived group of the target population with a behavioural and morbidity pattern fairly different from the general population. Secondly, in numerous low-income countries girls are frequently underrepresented in schools, which is definitely also the case for rural areas of Laos. However, the school-approach was justified for this study as enrolment rates are high for both sexes of urban and semi-urban children. Selection bias could still be a problem if children absent from school suffer from acute illness or they are absent for reasons making them different to their peers. It is worth noting that the response rate obtained was surprisingly high, maybe due to the fact that the examination team visited the schools over several days allowing children absent for only a short period to be included.

As for the clinical examination an attempt to avoid misclassification was made by training and calibration of the examiners and the standard of reliability set by WHO was achieved [14]. Meanwhile, the field examination took place with daylight being the only source of light and dental caries may thus be somewhat underreported. As regards risk behaviours, potential information bias like over reporting in favour of socially accepted behaviour such as tooth brushing may occur while underreporting could be the case for less accepted behaviour such as consumption of sugars. Moreover, recall bias especially in regards to food consumption and dental visits may be considered as well.

The relatively low mean DMFT found in this study is in line with those figures found in neighbouring China [8-10] and Thailand [7]. Having in mind the steep increase in sugar consumption in Laos one would have expected a higher DMFT compared to the countrywide DMFT of 2.0 found in 1991. Such trend was however not observed in the present study probably because the sugars consumption regardless of the steep increase is relatively low. The general sugars consumption in Laos is still far below the 15-20 kg/year observed as a threshold for a significant level of dental caries in a population [18]. It remains a challenge for Lao health authorities to preserve the consumption of sugars at a low level if caries is to be controlled effectively.

Sugars consumption is an important factor in the development of dental caries and the adverse effect is both related to the frequency and the amount of intake of free sugars [18]. The frequent intake of soft drinks observed among Lao students may possibly reflect the hot climate combined with the easy access to soft drinks during school hours. The present study has shown that the risk of dental caries is relatively high for children consuming sugary drinks. Such situation is found in several other low-income countries; for example, in Burkina Faso recent studies by Varenne et al found similar association between soft drink consumption and dental caries [5].

Having filled teeth was highly associated to urban location, having a literate mother, and having an advantaged socio-economic position while untreated decay was associated mainly to semi-urban location. These findings may indicate differences in access to health services and different levels of education on oral health. Significant variation in total caries was however only observed across socio-economic groups. Such differences may be related to the financial capacity of buying large amounts of sweets and snacks among the socio-economic advantaged groups.

A somewhat high number of children stated tooth brushing at least twice daily (unpublished report by the author). At the same time an extraordinary high prevalence of gingival bleeding was observed in the clinical investigation. This inconsistency could be explained by either over reporting of tooth brushing or simply reflecting a lack of tooth brushing skills. While the tooth brushing technique may be inadequate to the vast majority of the children, they may still gain some caries preventive effect of such practice when using toothpaste with appropriate level of fluoride. An over reporting of toothpaste use is likely and might explain the lack of difference in caries level between fluoride exposed and unexposed children. The low level of decay among frequent brushers could possibly mirror an effect of both mechanical brushing and fluoride effect.

The survey revealed a consistency between information on untreated dental caries and the subjective evaluation of own oral health. Similar findings have been reported from Brazil [19]. As for other low-income countries [6,20,21] untreated decay is the main contributor to the caries index in Lao children and does explain the various negative impacts on their quality of life. Due to the progressive development course of untreated caries the experience of pulp-involvement and pain is relatively common in spite of the harmlessly looking DMFT level. Untreated caries is highly associated with tooth ache and absenteeism is experienced significantly more frequent by socio-economic disadvantaged children compared to their better off peers. A socio-economic gradient among children in need for dental care does hereby have the potential to impact negatively on the learning capacity of the children and the health effect of education in general. Nevertheless, caries was found to be associated somewhat stronger to attitude level than to the knowledge level of the children.

The dominant dD-component reflects the lack of access to curative dental care in Laos and confirms this situation in most low income countries [1]. A high caries level was found to be associated with a recent dental visit and this mirrors the pattern observed in low-resource communities where dental visits are often prompted by pain and discomfort rather than regular preventive attendance [7,9,10,22-24]. The high mean of missing teeth among children with a history of dental visits might reflect either a radical treatment approach of the dentist or the fact that treatment is sought rather late by children leaving conditions of teeth beyond repair. Significantly less untreated caries would have been expected among the recently treated children but it appears that dental visits primarily are undertaken for pain relief rather than comprehensive treatment of the entire oral cavity.

Common risk factors may be responsible for associations between BMI and dental caries level and this assumption has been investigated in a number studies [25-28]. The results have however been inconclusive for several countries and this is echoed by the present study. Reasons for the lack of associations not only between dental caries and BMI but also between BMI and several other general health variables might be rather complicated due to the multi causal nature of obesity on the one hand and a population with little disease variation on the other hand.

## Conclusion

The observation of a relatively low DMFT in this survey of 12-year-olds should be seen as positive and it is well below the standard formulated for the year 2000 by the WHO [29]. In 1991, an oral health survey was undertaken [11], however, lack of information on the sampling method and criteria applied makes a direct comparison questionable. Therefore, time trend analyses of oral disease was not possible but could be provided for if a new survey would be conducted applying the principles of this survey.

The low level of dental caries provides a good starting point for oral health promotion and preventive activities. The challenge for health authorities will be continuously to keep the disease level low. In spite of the low level of dental caries the dentists in Laos are not sufficient in number to deal with the treatment need of the population leaving little time for preventive measures. Furthermore, dental health services in general have a strong curative focus and suffer from limited capacity to deliver population-based essential health care. The health authorities should therefore focus on planning and implementation of population-directed oral health promotion programmes through schools, in active collaboration with the education authorities. Schools play an important role by providing a health promoting environment and healthy lifestyles. For the prevention of dental caries access to food items, drinks and snacks rich in sugars should be discouraged and healthy choices have to be supported. Within schools, oral health education also should be oriented towards oral health self-care instrumental to prevention of dental caries and the poor gingival health of children. While the health personnel should provide technical input to such programme, the challenge of the educational system is to apply skills based learning and relate the rather theoretical oral health curriculum directly to the daily life of the children.

## Competing interests

The authors declare that they have no competing interests.

## Authors' contributions

NJ developed the protocol and design of the study, conducted the field work, analysed the data and was responsible for preparation of the manuscript. PEP assisted in the development of the protocol and design, supervised the data analyses and interpretation and contributed to the preparation of the manuscript.

Both authors have read and approved the final version of the manuscript.

## Pre-publication history

The pre-publication history for this paper can be accessed here:

http://www.biomedcentral.com/1472-6831/9/29/prepub
